# Fibrolysin in Pyloric Stenosis

**Published:** 1914-11

**Authors:** 


**Fibrolysin in Pyloric Stenosis.** J von Kovats, Budapest, "Med. Klin.," 1914, No. 11, reports a successful case, the pyloric obstruction apparently being relieved after the second injection, there being a gain of weight of 34 pounds in 2 months and no return of symptoms after 3½ years. He used in addition, the expedient of having the patient take considerable liquid with meals and then lie on the right side for 2 hours, to secure the influence of gravity.

